# *Juglans regia* Linn.: A Natural Repository of Vital Phytochemical and Pharmacological Compounds

**DOI:** 10.3390/life13020380

**Published:** 2023-01-30

**Authors:** Aeyaz Ahmad Bhat, Adnan Shakeel, Sadaf Rafiq, Iqra Farooq, Azad Quyoom Malik, Mohammed E. Alghuthami, Sarah Alharthi, Husam Qanash, Saif A. Alharthy

**Affiliations:** 1 Department of Chemistry, Lovely Professional University, Phagwara 144411, India; 2 Department of Botany, Aligarh Muslim University, Aligarh 202002, India; 3 Division of Floriculture and Landscape Architecture, Sher-e-Kashmir University of Agricultural Sciences and Technology of Kashmir, Srinagar 190025, India; 4 CSIR—Indian Institute of Integrative Medicine, Jammu 180001, India; 5 GCC Accreditation Centre GAC, AL Safarat, Riyadh 12511, Saudi Arabia; 6 Center of Advanced Research in Science and Technology, Taif University, P.O. Box 11099, Taif 21944, Saudi Arabia; 7 Department of Chemistry, College of Science, Taif University, P.O. Box 11099, Taif 21944, Saudi Arabia; 8 Department of Medical Laboratory Science, College of Applied Medical Sciences, University of Ha’il, Hail 55476, Saudi Arabia; 9 Molecular Diagnostics and Personalized Therapeutics Unit, University of Ha’il, Hail 55476, Saudi Arabia; 10 Department of Medical Laboratory Sciences, King Abdulaziz University, P.O. Box 80216, Jeddah 21589, Saudi Arabia; 11 Toxicology and Forensic Sciences Unit, King Fahd Medical Research Center, King Abdulaziz University, P.O. Box 80216, Jeddah 21589, Saudi Arabia

**Keywords:** antibacterial, antifungal, antioxidant, anticancer, juglone, *Juglans regia*

## Abstract

*Juglans regia* Linn. is a valuable medicinal plant that possesses the therapeutic potential to treat a wide range of diseases in humans. It has been known to have significant nutritional and curative properties since ancient times, and almost all parts of this plant have been utilized to cure numerous fungal and bacterial disorders. The separation and identification of the active ingredients in *J. regia* as well as the testing of those active compounds for pharmacological properties are currently of great interest. Recently, the naphthoquinones extracted from walnut have been observed to inhibit the enzymes essential for viral protein synthesis in the SARS-CoV-2. Anticancer characteristics have been observed in the synthetic triazole analogue derivatives of juglone, and the unique modifications in the parent derivative of juglone have paved the way for further synthetic research in this area. Though there are some research articles available on the pharmacological importance of *J. regia*, a comprehensive review article to summarize these findings is still required. The current review, therefore, abridges the most recent scientific findings about antimicrobial, antioxidant, anti-fungal, and anticancer properties of various discovered and separated chemical compounds from different solvents and different parts of *J. regia*.

## 1. Introduction

*Juglans regia* Linn. belongs to the family Juglandaceae and is an aromatic transient tree that grows in abundance in the North-Western Himalayas of Kashmir that produces most of the world’s walnuts, accounting for around 88% of total walnut production [1]. The bark of the tree is gray, and the tree has longitudinally fissured leaves that are alternating, imparipinnate, sessile, elliptic, or oblong-lanceolate. Flowers are unisexual and there are around two genera and fifteen species in the family. The leaves are evenly organized, measuring 25–40 cm in length, unevenly pinnate with five to nine leaflets and placed in a regular pattern. The medicinal plant *Juglans regia* Linn. has been used extensively in traditional medicine for a variety of illnesses, including helminthiasis, diarrhea, sinusitis, stomach aches, arthritis, asthma, eczema, scrofula, skin disorders, and various endocrine diseases such as diabetes mellitus, anorexia, thyroid dysfunctions, cancer, and infectious diseases [2,3]. The green fruit ripens in the autumn, when the entire fruit, including the husk, drops from the tree. The huge seed contains a rich flavor and a thin edible shell.

**Figure 1:**
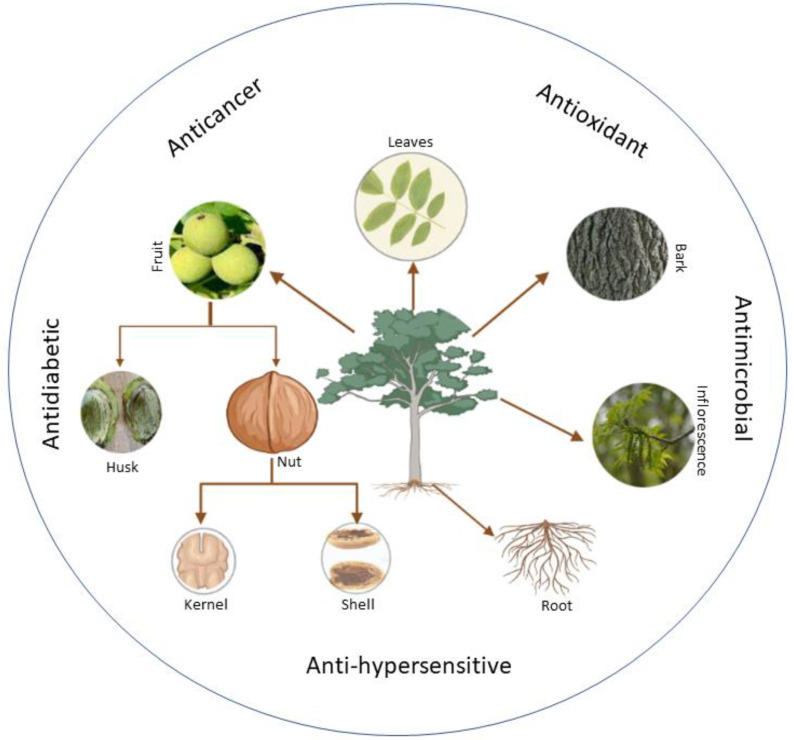
Different parts of *Juglans regia* with medicinal properties.

The phytochemistry of the tree has extensively been studied and many important phytochemical activities have been exploited [3]. However, the number of elements may vary from one species to another in a different place based on several parameters, such as geographical location, time, temperature, genetic makeup, and other factors. Several studies on the phytochemical examination of the tree’s various components with numerous health benefits have been performed (Figure 1 and Table 1). According to these studies, the chemical components found in walnuts fluctuate depending on the climate. The oil is rich in polyunsaturated fatty acids, tocopherols and phytosterols. The walnut fruits are valuable, and tasty walnut leaves contain many chemicals, the most prominent of which are Aesculin, Taxifolin-pantocid, Quercetin-glucronide, Kaempferol-rhamnoside, Syringetin-O-Hexoside, Myricetin-3-O-glucoside, Myricetin-3-O-pantocid, and Epicatechin [3,4].

## 2. Traditional and Ethnobotanical Uses

For ages, the plant has been utilized in tropical medicine to treat cutaneous irritation and excessive perspiration in the hands and feet [44]. The leaves are traditionally used to treat sinusitis and stomach aches and are also used throughout the world as an antibacterial, anthelmintic, antidiarrheal, hypoglycemic, tonic, and depurative medicine [45,46]. In Turkish traditional medicine, fresh leaves are applied to the naked body or the forehead to reduce fever or to swollen joints to treat rheumatic agony [47]. The wood is durable and perfect for furniture and contains essential oils for aromatic essence. As a supplementary ointment, the leaves of this plant are applied directly to cure sunburn, superficial burns, scalp itching, dandruff, and other skin disorders [48]. The plant has been used to treat inflammatory diseases, diabetes, and cardiac illness in Palestine [49] as well as to help older men’s vascular and prostate health [50]. In addition to using bark and unripe fruit for piscicidal action, the Lotha, Angami, and Sumi tribes of Kohima (Nagaland) also employed leaves of *J. regia* as astringents, anthelmintics, and treatments for dermatitis [51].

## 3. Pharmacological Applications

The use of medicinal herbs has increased substantially in recent years, resulting in the utilization of both traditional remedies and medicinal plants for the novel drug discoveries, and the development of more effective pharmaceuticals and nutraceuticals with fewer side effects. The main focus of this review is to explore the pharmacological applications, phytochemistry, and the therapeutic benefits of the plant extracts on antimicrobial, antioxidant, anticancer, and anti-inflammatory activities and many others (Table 2).

### 3.1. Antibacterial Activity

**Figure 2:**
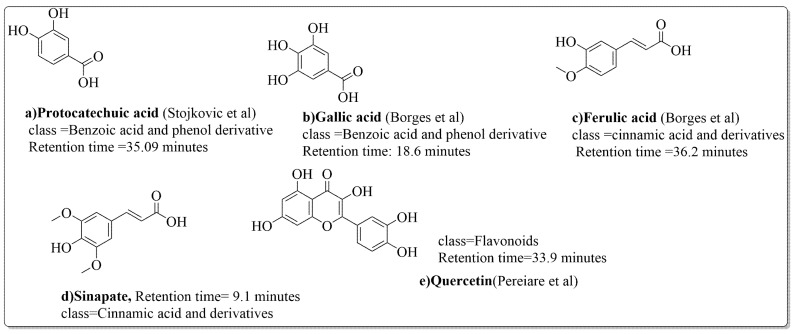
Phenolic compounds having antibacterial activities from pellicle extract of *J. regia*, Nowicki et al. [69].

The disc diffusion method (zone of inhibition) was used to identify and examine the antibacterial activity of *Juglans regia* hull extracts and it was revealed that these extracts possessed an appreciable antibacterial activity against a variety of bacterial species, including *E. coli, B. subtilis, K*. *aerogene,* and *S. aureus* [67]. The findings also implied that *J. regia* green hull extract could be useful in the treatment of acne due to the virtue of having anti-inflammatory effects. Some leaf extracts of *J. regia* showed the ability to prevent the growth of *K. pneumonia* at minimum inhibitory concentrations (MIC) of 100 mg/mL [68]; other extracts from the hull prepared in different solvents were able to stop the development of *P. aeruginosa* and *E. Coli* at MIC of 50 and 100 mg/mL, respectively [69]. Several antibacterial chemicals identified from *Juglans regia* pellicle are represented (Figure 2).

**Figure 3:**
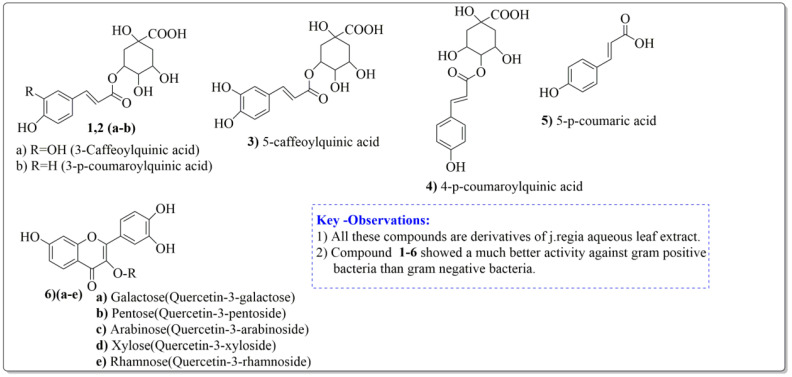
Extracted phenolic compounds obtained from *J. regia*, Rahman et al. [70].

Pereira et al. [70] investigated the antibacterial activity of aqueous extracts from *Juglans regia* leaves against Gram-positive and Gram-negative microorganisms. The aqueous extracts mostly contained phenolic compounds which were obtained by shade drying the leaves, followed by the extraction of the compounds with various solvents, such as ether, alcohol, and water. By using HPLC-DAD, phenolic substances were identified and quantified. The chromatograms obtained at 320 and 350 nm revealed the presence of the different compounds (Figure 3, compounds 1-6). The obtained extracts were screened against Gram-positive (*B. subtilis*, *Staphylococcus aureus*, *Bacillus cereus*) and Gram-negative (*Escherichia coli*, *Pseudomonas aeruginosa*, *Klebsiella pneumoniae*) bacteria which revealed that Gram-positive bacteria development was inhibited to a greater extent than the inhibition in the case of Gram-negative bacteria, clearly indicating the fact that the ether, alcohol, and water extracts turned out to be favorable for the inhibition activity against Gram-positive bacteria. It was also observed that *B. cereus* was the most suspectable microorganism at MIC of 0.1 mg/mL. The order of inhibition of the above-made extracts from different solvents was found to be *B. cereus > S. aureus > B. subtilis*.

The antibacterial characteristics of the essential oil from the leaves and its constituents have been examined by Rather et al. [71]. In this study, the essential oil was extracted from the leaves of *J. regia* and analyzed using GC-MS, yielding a total of 38 compounds, accounting for 92.7% of the oil substance, including major components such as β-pinene (30.5%), α-pinene (15.1%), germacrene D (14.4%), β-caryophyllene (15.5%), and Limonene (3.6%). Disc diffusion and microdilution methods were used to test all essential oils and individual constituents for antibacterial activity against a group of Gram-positive (*B. subtilis* MTCC-441, *S. epidermidis* MTCC-43, *S. aureus*) and Gram-negative (*B. subtilis* MTCC-441, *S. epidermidis* MTCC-435, *P. vulgaris* MTCC-321, *Salmonella typhi*, *Pseudomonas aeruginosa* MTCC-1688, *Klebsiella pneumonia*, *Shigella dysenteriae,* and *Escherichia coli*) bacteria. It should be noted that the essential oil and its components were effective against all bacterial strains screened for antibacterial activity with a wide range of activity. However, it was discovered that Gram-positive bacteria were found to be more inhibited compared to Gram-negative bacteria in the development and cell division processes. The potent compounds at the maximum inhibition category include α-pinene, β pinene, β-caryophyllene, and Germacrene D. For the most inhibited Gram-positive bacteria (*S. epidermidis*, *B. subtilis and S. aureus*), the MIC recorded were 15.62, 15.62, and 15.62 g/mL for *J. regia* essential oil, 48.31, 47.21, and 45.62 g/mL for α-pinene, 46.55, 46.55, and 41.33 g/mL for β-pinene, respectively, clearly indicating the fact that the essential oil present in the leaves of the plant played a key role in the inhibition of different bacteria. Gentamicin had the lowest potency in this category, with MIC values of 5.95, 5.90, and 3.90 g/mL, respectively, for the above said Gram-positive bacteria. The findings indicate that these three bacterial species are the most susceptible to essential oils and components. The changes in the activity against different bacteria might be ascribed to structural differences between the microbes and the different developmental stages in different bacteria [71,72].

### 3.2. Antioxidant Activity

**Figure 4:**
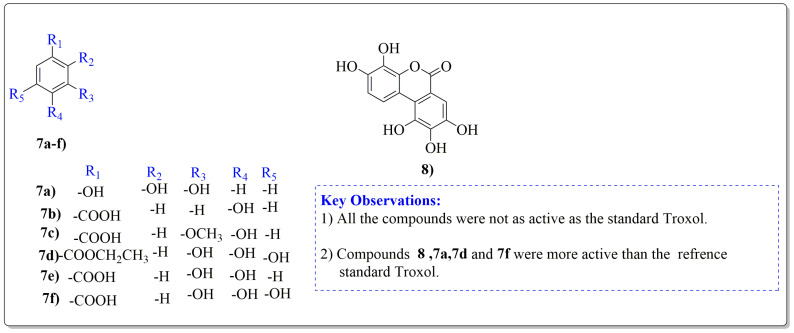
Phenolic antioxidant compounds from walnut kernels, Zhai et al. [76].

*Juglans regia* leaves are high in flavonoid content, which has been linked to regulating immunological function and boosting anticancer activity, which is a global threat nowadays [73]. The antioxidant potential of walnut kernels, husks, and leaf extracts formed in different solvents, such as ethyl acetate, butanol, methanol, ether, and aqueous methanol, have been evaluated using a variety of methods. Some common and highly placed methods include the reducing power method, lipid oxidation inhibition method, and DPPH radical scavenging activity. Moreover, various studies have validated the antioxidant activity of the flavonoids present in leaves and fruit of *J. regia* and it was established that almost all the extracts prepared in different solvents possessed this property [74,75]. Antioxidant phenolic components from *J. regia* walnut kernels, which were extracted and fractionalized based on the principle of polarity and interactions with the solvent system used (Petroleum ether, Ethyl-acetate, n-butanol, and aqueous solvent) and on the basis of which they were designated as petroleum ether fraction (PEF), ethyl-acetate fraction, n-butanol fraction, and others [76]. Column chromatography over silica gel eluted with increasing polarity was used to separate the more active fractions (EEF and BUF) (Figure 4).

All the derivative compounds (7a–f and 8) were screened for α, α-diphenyl-β-picrylhydrazyl (DPPH) free radical scavenging activity using Trolox IC_50_ = 0.026 mM as a reference standard. The IC_50_ values revealed that the EEF and BUF fractions with (IC_50_ = 0.83 and 0.88 µM) were the most active in terms of DPPH free radical scavenging among all the used fractions (PEF, EEF, BUF, and AF), as described earlier. Compounds 8, 7f, 7d, and 7a, however, demonstrated considerable DPPH scavenging capabilities as depicted by IC_50_ values of (0.007, 0.011, 0.013, and 0.015 µM), respectively, indicating that they were more active than the reference standard Trolox (IC_50_ = 0.026 µM). For the first time, spectroscopic methods were used to isolate and identify seven phenolic compounds with significant antioxidant activities in *J. regia*: pyrogallol (7a), p-hydroxybenzoic acid (7b), vanillic acid (7c), ethyl gallate (7d), protocatechuic acid (7e), gallic acid (7f), and 3,4,8,9,10-pentahydroxydibenzo [b, d] pyran-6-one (8). The DPPH scavenging capacity of these compounds was ranked in the following order: 7 > 6 ≥ 4 ≥1 > Trolox ≥ 5 > 3 > 2. The findings of this study suggested that the number of hydroxyls in these phenolic compounds’ aromatic rings may influence their antioxidant activities. Some important structure-activity relationships of the derivative compound (7f) indicate the essence of the COOH group as R_1_ with other groups as R_3_ = R_4_ = R_5_ = OH. This activity can be enhanced in the near future research by introducing some new acid electron withdrawing groups (EWG) as the replacement of R_1_ in the said derivative (7f), e.g., introduction of carbonyl, thiol, and aldehyde. The antioxidant activity, which also showed a sharp increase in case of the derivative (7d), can be reasonably attributed to the presence of R_1_ = COOCH_2_CH_3_ group with the aid of R_3_ = R_4_ = R_5_ = OH in the moiety.

*J. regia* leaf extracts are effective scavengers of pro-oxidant reactive species and can be used as a readily available source of natural antioxidants. The growing interest in replacing synthetic food antioxidants with natural ones has fueled research on vegetable sources and raw material screening in the search for new antioxidants. An ethanol:water (4:6) extract of Juglans regia leaves was evaluated in vitro for its putative scavenging effects on reactive oxygen species (ROS) [hydroxyl radical (HO**.**), superoxide radical (O_2_^−^, peroxyl radical (ROO^.^), hydrogen peroxide (H_2_O_2_**.**)] and reactive nitrogen species (RNS) [nitric oxide (NO^.^), and peroxynitrite anion (ONOO^−^)].The extract demonstrated strong scavenging activity against all reactive species tested, with all IC_50s_ found at the lg/mL level. The IC_50s_ (mean ± SE) for ROS O_2_^−^ and H_2_O_2_ radical were found to be 47.6, 4.6 and 383, 17 lg/mL, respectively. In addition, the antioxidant activity discovered in this study can be used to validate the use of *J. regia* leaf extracts in the treatment of inflammatory diseases. The current study found that the *J. regia* leaf can be used as a readily available source of natural antioxidants. The extract under investigation could aid in the prevention of lipid peroxidation as well as the protection of food, excipient bases, and medicines from oxidative damage. Furthermore, the observed antioxidant activity can at least partially justify the use of *J. regia* leaves as a therapeutic agent in inflammatory diseases. Nonetheless, its potential toxicity should be addressed before any practical application. [77].

By using DPPH and hydroxyl radical scavenging assays, Zurek et al. [78] investigated the antioxidant activity of the leaf’s essential oil and some of its constituents. The antioxidant activity was measured using the 2,2-diphenyl-1-picrylhydrazyl (DPPH) radical scavenging activity assay, the copper ion reduction assay (CUPRAC), metal chelating ability (ChA), and the ability to scavenge superoxide (O2^−^) and hydroxyl (OH^−^) radicals. These methods are regarded as important indicators of plant samples’ antioxidant potential. The free radical scavenging DPPH had the lowest IC_50_ value in this study, corresponding to the highest antioxidant activity. The calculated half maximum inhibitory concentration (IC_50_) value was 22.34, 2.70 g/mL, which was higher than the positive control ascorbic acid (5.00, 0.01 g/mL). The chelating capacity test (71.69, 0.02 g/mL), superoxide scavenging (147.06, 0.27 g/mL), and hydroxyl radicals test (41.85, 0.09 g/mL) all had low IC_50_ values, indicating high antioxidant activity. The findings revealed that *J. regia* flowers were found to have a high ability to scavenge free radicals, which could be attributed to their high content of bioactive compounds. Furthermore, the results revealed a strong relationship between the phenolic compounds studied and their antioxidant activities.

### 3.3. Analgesic and Anti-Inflammatory Properties

*J. regia* extracts from aqueous (2.87 and 1.64 g/kg) and ethanolic (2.044 and 1.17 g/kg) solutions have demonstrated antinociceptive effectiveness in a hot plate test, according to Hosseinzadeh et al. [79]. The hot-plate test was performed on eight male and female mice groups. The metal surface temperature was kept constant at 55 °C. Before and after drug administration, the latency to a discomfort reaction (licking paws or jumping) was measured. 30 min after the extracts were given to groups of eight male and female mice, they were given an intraperitoneal injection of 0.7% *v*/*v* acetic acid solution (volume of injection 0.1 mL/10 g body wt.). The number of writhing produced in these animals was counted for 30 min after acid injection. The anti-inflammatory activity against acute inflammation was evaluated in mice using the xylene-induced ear edema method. The mice were divided into eight groups. 30 min after the different doses of extract were injected intravenously, diclofenac and 0.03 mL of xylene were applied to the anterior and posterior surfaces of the right ear. The left ear was used as a control. The mice were sacrificed two hours after xylene application, and both ears were removed. Circular sections were excised and weighed using a cork borer with a 9 mm diameter. The weight gain caused by the irritant was calculated by subtracting the weight of the untreated left ear section from the weight of the treated right ear section. In mice, the anti-inflammatory activity against chronic inflammation was assessed using the cotton pellet granuloma method. The 30 mg dental cotton pellets were sterilized in an air oven at 121 °C for 20 min before being impregnated with 0.4 mL of an ampicillin aqueous solution. Under ketamine (65 mg/kg body weight) and xylazine (6.5 mg/kg body weight) anesthesia, two cotton pellets, one on each side, were implanted subcutaneously in the shoulder region of mice. The extract and diclofenac were administered once daily for seven days. The rats were killed on day 8, and the pellets and surrounding granulation tissue were dried at 60 °C for 24 h. In mice, the intraperitoneal LD_50_ values of *J. regia* aqueous and ethanolic leaves extract were 5.5 g/kg (4.1–6.5) and 3.3 g/kg (3.1–3.5), respectively, with maximum nonfatal doses of 4.1 g/kg and 2.93 g/kg. However, it should be noted that both extracts displayed an anti-inflammatory effect in xylene at lower dosages. The extracts demonstrated antinociceptive action in the Writhing test that was not inhibited by naloxone (lifesaving medication). The extracts were found to have anti-inflammatory properties in the case of chronic inflammation. *J. regia* leaves have been shown to have an antinociceptive action via non-opioid receptors as well as an anti-inflammatory impact against acute and chronic inflammation, making them an effective drug with analgesic and anti-inflammatory properties against rheumatoid arthritis [80].

### 3.4. Antidepressant Activity

Depression has been classified as a mood disorder and has been defined as a feeling of sadness, loss, or anger [81]. Treatments derived from the *Juglans regia* L. flower and its leaf extracts have been found to be highly successful in treating depression. The effects of the forced swimming test (FST) and tail suspension test (TST) in mice were quite evident [82].

#### 3.4.1. Forced Swimming Test

The immobility time was calculated by observing the muscle movements of rats that were placed in a pool of water. A glass cylinder with a diameter of 25 cm and a height of 23 cm was filled with water to a height of 12 cm. The water temperature was 23 °C. Each rat was given a single injection of the extract. The animals were tested thirty minutes later. For the first two minutes, each animal was given time to get used to the new circumstances before having their immobility time recorded. For the following six minutes, conditions of increased muscle control interrupted with periods of immobility. For the next four minutes, immobility was timed using a stopwatch.

#### 3.4.2. Tail Suspension Test

The tail-suspension test was the second method used to evaluate the extract’s antidepressant effect. Rats were tested thirty minutes after a single drug or vehicle injection. A cord which was about 50 cm long was stretched between two metal tripods at a height of 70 cm, to which the rats were taped by the tail. The rats became immobile after a period of vigorous motor activity, and the time was recorded with a stopwatch for a total of 4 min. When rats hung passively and completely motionless, they were considered immobile.

The mean duration of immobility in the control group was 188.33, 2.16 s, whereas it was 151.16, 2.56 s, in the fluoxetine group. The reduction in immobility was found to be statistically significant (*p* < 0.05). The decrease in immobility in the extract groups was also significant (*p* < 0.05) for both doses. For 100 mg/kg and 150 mg/kg body weight, the total duration of immobility was found to be 160.66, 3.76 s and 154.83, 4.32 s, respectively. Regarding the effect on immobility in the tail suspension test, in the control group, the mean duration of immobility was 193.33, 1.96 s, whereas in the fluoxetine group, it was 147.16, 2.48 s. The reduction in immobility was found to be statistically significant (*p* < 0.05). The total duration of immobility for 100 mg/kg body weight was found to be 168.39 s and 148.66 1.75 s for 150 mg/kg body weight. The decrease in immobility in the extract groups was also significant (*p* < 0.05) for both doses [83]. This remarkable antidepressant activity was attributed to the phenol and flavonoid contents, especially quercetin. In addition, the presence of omega-3 fatty acids in *Juglans regia* fruit extracts may have depressive properties [84]. More research is needed to fully understand the mechanism of antidepressant activity.

### 3.5. Antiviral Activities

**Figure 5:**
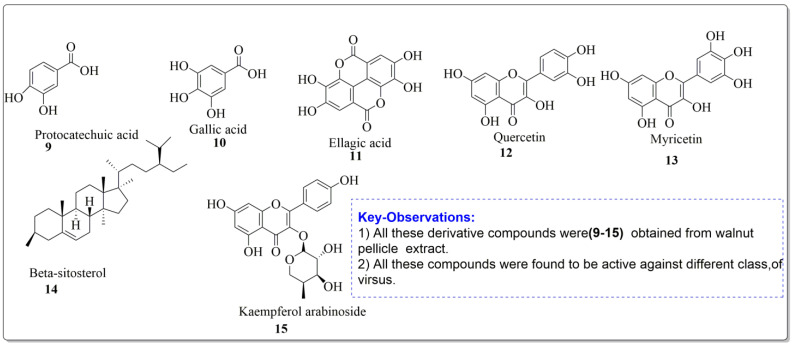
Compound derivatives from walnut pellicle extract, Alkhawajah et al. [87].

Mouhajir et al. [85] studied the activity of *Juglans regia* methanol extracts. At noncytotoxic concentrations, the 2 mg/mL concentration was tested against Sindbis virus (SINV), herpes simplex virus (HSV) and poliovirus. Vardhini [86] also used a computational method to investigate juglone’s antiviral activities and the results were well supported by molecular docking studies. In this study, the ligand which had the highest binding affinity had a dock score of 114.967 against ASP 29, ASP 30, and ASP 30, which are the hydrogen bonds that existed between the ligand and the protein molecule. Using phytochemical and chromatographic methods to separate components from *J. regia*, other researchers [85] investigated the impact of anti-HIV activity in vitro. The impact of anti-HIV activity in vitro was assessed using MT4 cells and HIV-III B virus. The target research was conducted using BIACORE 3000 molecules linked equipment. Angeli et al. [87] studied the walnut pellicle extracts and derived some handful number of antiviral compounds which possessed the inhibitory activity against HSV-1 and HSV-2 replication. The ID_50_ (con. which inhibited 50% virus formation) were found to be 10 and 8 µg/mL for HSV-1 and HSV-2, respectively. However, the walnut pellicle extract was ineffective against Echovirus9 (ECHO-9), Poliovirus1 (polio1), Coxsackievirus B1 (coxsackie B1) and Adenovirus (Adenoid). The compounds extracted from walnut pellicle extract were found to be active against viral diseases (Figure 5).

The global outbreak of COVID-19 was caused by SARS-CoV-2, a positive-sense single-stranded RNA coronavirus. The virus’s main protease (Mpro), the major enzyme processing viral polyproteins, contributed to SARS-CoV-2 replication and transcription in host cells and has been identified as an appealing target in drug discovery. A series of 1,4-naphthoquinones with juglone (compound isolated from leaves of walnut) skeletons were synthesized and tested for inhibitory efficacy against SARS-CoV-2 Mpro. At a concentration of 10 µM, more than half of the tested naphthoquinones effectively inhibited the target enzyme, with an inhibition rate of more than 90%. The characteristics of substituents and their position on the juglone core scaffold were identified as key ingredients for enzyme inhibitory activity in the structure-activity relationships (SARs) analysis. The most active compound, 2-acetyl-8-methoxy-1,4-naphthoquinone, had an IC_50_ value of 72.07–4.84 nM against Mpro-mediated hydrolysis of the fluorescently labelled peptide, which was much higher than shikonin as the positive control. In molecular docking studies, it fit well into the active site cavity of the enzyme by forming hydrogen bonds with adjacent amino acid residues. The results of in vitro antiviral activity testing revealed that the most potent Mpro inhibitor could significantly suppress SARS-CoV-2 replication in Vero E6 cells at low micromolar concentrations, with an EC_50_ value of about 4.55 µM. Under the conditions tested, it was non-toxic to the host Vero E6 cells. The current study suggested that the juglone skeleton could serve as a primary template for the development of potent Mpro inhibitors [88].

#### Enzyme Inhibition Mechanism

The inhibitory activity of the prepared quinones against Mpro of SARS-CoV-2 was evaluated using a fluorescently labelled short peptide containing a Q-S scissile bond. In the first library of compounds, we tested the enzymatic inhibition rate of several naturally occurring naphthoquinones (juglone 2, 7-methyl juglone, lawsone, plumbagin and shikonin), 9,10-anthraquinones (emodin, rhein and aloe emodin), and synthetic vitamin K3 against SARS-CoV-2 Mpro at 10 µM. The primary screening results showed that the majority of the natural quinones were ineffective, with inhibition rates of less than 10% at 10 µM. Vitamin K3 was also inactive, with a 12.7% inhibition rate. The positive control was the natural naphthoquinone shikonin, which had previously been identified as a strong Mpro inhibitor (IC_50_ = 15.75 8.22 µM). At a concentration of 10 M, it had a moderate inhibitory effect on the target enzyme. In the first library of naphthoquinones, juglone and 7-methyl juglone inhibited Mpro completely, resulting in the loss of its hydrolytic efficacy. The lead compounds for further structural modifications were the two natural naphthoquinones. In the second library, juglone and 7-methyl juglone derivatives were synthesized by adding a few groups to their naphthoquinone scaffold and modifying the phenolic hydroxyl group. The results indicated that almost all of the juglone derivatives in the second library retained their high inhibitory potency at concentrations of 10 M and 1 M. A few analogues exhibited significantly higher potency than the parent compounds juglone and 7-methyl juglone at 0.1 µM. The compounds with an enzymatic inhibition rate of more than 25% at 0.1 µM concentration were then subjected to IC_50_ value screening.

### 3.6. Antidiabetic Activity

*J. regia* leaf extract has been thought to be advantageous for the treatment of diabetes mellitus since ancient times, and this notion has been scientifically proven to be effective. Blood glucose, glycosylated hemoglobin, LDL, lipid, and cholesterol levels were found to be reduced by alcohol extracts from the plant’s leaves. Streptozotocin-treated rats were administered by treating with 200 and 400 mg/kg leaf extracts of *J. regia* leaf extract for 28 days, and it alleviated hyperglycemia through reduced glycosylated hemoglobin and enhanced insulin sensitivity [89]. They looked at how *J. regia* leaf extract affected hyperglycemia in type 2 diabetic patients. The leaves of *J. regia* were initially freshly harvested, then washed and dried at 25 °C temperature in the shade before being powdered, and the powder was extracted at room temperature using the percolation technique and 70% aqueous ethanol. The solvent was eliminated using Whatman paper filters and the crude extract was standardized by counting the total phenol concentration after evaporating at a temperature of no more than 40 °C under decreased pressure. Later, capsules of *Juglans regia* and a placebo with the same appearance were created. The *J. regia* capsules included 100 mg of leaf extract powder. In the manufacture of the leaf extract powder, toast powder was chosen as the placebo and as the excipient. The research was conducted on a group of individuals who were divided into two groups: those who had received *J. regia* and those who had received a placebo. The levels of the constituents fasting blood glucose (FBG), HbA1c, total cholesterol, and triglyceride were measured in *J. regia*-treated patients and they were found to be on a lower side than those in the placebo group, with no adverse effects. Finally, for type 2 diabetes, three months of treatment with 100 mg of *Juglans regia* leaf extract twice daily improved glucose control with no noticeable side effects. The results showed that FBG, HbA1c, total cholesterol, and triglyceride levels in Juglans regia-treated patients were significantly lower than in the baseline and placebo groups. When compared to the placebo group, patients in the Juglans regia group were significantly more satisfied with their treatment. Except for more GI events (especially mild diarrhea) associated with extract treatment at the start of the study, no liver, kidney, or other side effects were observed in the groups.

Testicular dysfunction is a complication of diabetes, and *Juglans regia L*. leaf extract contains phenolic compounds with hypoglycemic and antioxidative properties. Nasiry et al. investigated whether *J. regia* leaf extract could protect against the negative effects of diabetes on oxidative stress, testis histology and testosterone hormone production [90]. In their research, four groups of male rats were used: a control (nondiabetic) group given saline, a diabetic group, a diabetic + *J. regia* group that received *J. regia* leaf extract and a *J. regia* group (nondiabetic) that received *J. regia* leaf extract only. They looked at histopathological and histomorphometric changes, serum testosterone, malondialdehyde (MDA), glutathione (GSH), superoxide dismutase (SOD), and catalase (CAT) levels to see how *J. regia* L. leaf extract affected testicular functions in diabetic animals. In the testis of diabetic rats, there was a reduction in MDA as well as an improvement in antioxidant status; *J. regia* leaf extract attenuated these abnormalities. In diabetic rats, it was found that there was a significantly decrease in the levels of testosterone, GSH, SOD, and other antioxidant biomarkers; these levels were restored after the *J. regia* leaf extract was administrated. After administering the *J. regia* leaf extract, the MDA level and improved antioxidant status in the testis of diabetic rats were found. Because of its antioxidant, anti-inflammatory and anti-apoptotic properties, *J. regia* leaf extract may have protective effects against diabetic dysfunction in the testis, according to the findings.

### 3.7. Anticancer Activity

Cancer treatment has long been a difficulty for medical science, and research into a variety of medicinal plants has resulted in a potential treatment for the disease’s early stages [91,92]. Even while cancer has been identified as a burden on human civilization, no comprehensive cure has yet been established [93]. Juglone has been demonstrated to inhibit intestinal carcinogenesis in mice, suggesting that it might be a potential chemo preventive medication for neoplasia in human intestines [94]. The human carcinoma cells lines HCT-15 cells, HL-60 cells, and doxorubicin-resistant HL-60R cells have all demonstrated the potency of juglone as a strong cytotoxin [95]. Human cancer renal cell lines A-498 and 769-P, as well as the colon tumor cell lines Caco-2, were inhibited in a concentration-dependent way by walnut methanolic extract out from the seeds, greenish husks, and leaves of *Juglans regia*. All extracts inhibited the growth in 769-P renal and Caco-2 colon cancer cells (IC_50_ values of 0.352 and 0.229 mg/mL, respectively; range, 0.226 to 0.29 mg/mL), but the walnut extract of leaves was more effective at suppressing cell proliferation than green husks and extracts of seeds (IC_50_ values of 0.352 and 0.229 mg/mL, respectively).

**Figure 6:**
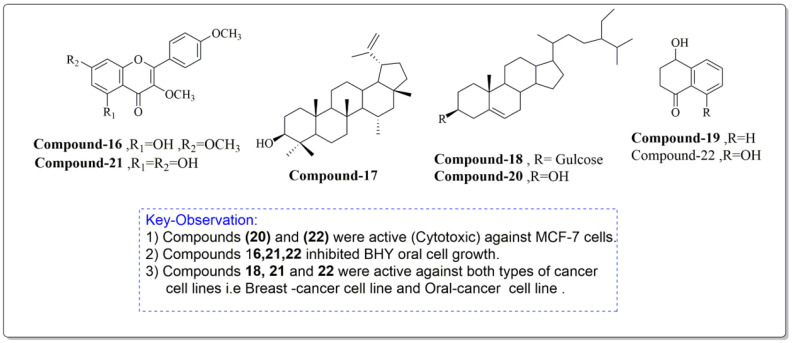
Compounds having antiproliferative and apoptotic activity against MCF-7 and BHY cancer cell lines, Noumi et al. [95].

Constituents of *J. regia* chloroform leaf extract was tested for anti-proliferative and apoptotic effects on human breast (MCF-7) and oral tumor (BHY) cell lines. The components were extracted from shrub leaves which were air dried, pulverized, and then percolated in n-hexane for 24 h. After three days of extraction, all extracts were filtered and dried with a rotatory evaporator. At a lower pressure, the solvent, n-hexane, was extracted. The remaining powder was then suspended and extracted to obtain the chloroform fraction, which was further purified using chromatography (Figure 6). The proliferative and apoptotic activities of the compounds mentioned above (16–22) were investigated. Derivatives (20 and 22) were found to be significantly cytotoxic to MCF-7 cell lines, whereas compounds (16, 21, 22) significantly inhibited BHY cell proliferation. According to the IC_50_ values, MCF-7 cell lines were also the most sensitive to virtually all chemicals. Compounds (21) (IC_50_ = 50.98 µM) and (22) (IC_50_ = 21.30 µM) were selectively active against both cancer cell lines, MCF-7 and BHY, but were significantly less effective against normal cells. The compounds (16–22) suppressed cell population development in human tumor cell lines MCF-7 and BHY, as well as mouse fibroblast cell lines, using the MIT test at 24, 48, and 72 h (NIH-3T3). However, the best proliferation activities were obtained after 72 h. It is worth mentioning that compounds 21 and 22, which are plant flavonoids, and a plant naphthoquinone can be further studied well to explore their new biological activities such as antidiabetic and antibacterial activities. Moreover, these compounds induced apoptosis in MCF-7 cell lines by the well-known mechanism caspase-3 independent pathway [95].

**Figure 7:**
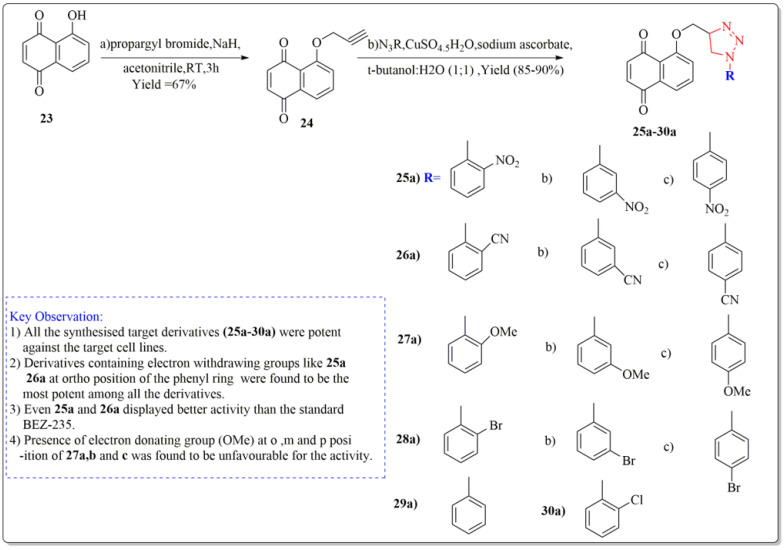
Synthesis of modified *juglone* derivatives (triazole analogs) as cytotoxic agents, Haque et al. [96].

One of the important synthetic breakthroughs was given by Zhang et al. [96] as they developed modified Juglone from *J. regia* as a strong cytotoxic drug against carcinoma cell lines of lungs by isolating and modifying the compound. The synthesis started with a solution of Juglone (23) in acetonitrile (isolated from *J. regia* roots). At 20 °C, the solution was portioned with NaH and agitated continuously for 10 min. Propargyl bromide was added drop by drop, and the suspension was again agitated for two hours. Ethyl acetate was used to extract the reaction mixture, which was then purified on a silica gel column to get the derivative (24). The derivative (24) was then treated with organic azide, sodium ascorbate, water:butanol (1:1), and copper ) (ii) pentahydrate to yield the final target derivatives (25a–30a). Juglone derivates having cytotoxic effects are depicted in Figure 7. All the synthesized target derivatives (25a–30a) were evaluated through cytotoxic assays against lung cancer cell lines (NCl-H322 and A549) using BEZ-235 (IC_50_ = 9.80 µM) as a positive control. Among all the synthesized derivatives of Juglone, 25a and 26a bearing o-nitro and o-cyano phenyl moieties, respectively, displayed the most potency (IC_50_ = 4.72, 8.90 and 4.67, 7.94 µM) against both the lung cancer cell lines (NCl-H322 and A549), respectively. Both these derivatives even displayed better activity results than the standard positive control BEZ-235 (IC_50_ = 9.80 µM). Derivatives 27a, 27b, and 27c with ortho, meta, and para-methoxy R moieties, respectively, were very less potent towards the cancer cell lines. Similarly, derivatives (28 a, b, c) bearing o, m, and p-bromophenol moieties were lesser active than juglone (IC_50_ = 19.32 µM) and standard BEZ-235 as displayed by the IC_50_ values (24.34, 21.82, 24.55 µM), respectively. Moreover, derivatives 29a and 30a bearing a phenyl and o-chlorophenyl displayed slight better activity than juglone; however, they were less potent than the standard BEZ-235 against the lung cancer cell lines (NCl-H322 and A549). From the above results, it can be clearly stated that the introduction of EWG at ortho position of the phenyl ring in the said derivative (25a, 26a) turned out to be favorable for the net potency of the derivatives, which can be enhanced in the new research by introducing some other EWG’s at ortho position of the phenyl ring, such as the EWG group carbonyl, carboxyl, CF_3_, or fluorine at ortho position. Moreover, from structure activity relationship (SAR) studies, it is clear that changing the position of groups from ortho to meta in (25b and 26b) or from ortho to para in (25c and 26c) in the above structures showed a dramatic decrease in the activity as depicted by the IC_50_ values of 25b, 26b, 25c, 26c (IC_50 =_ 15.6, 17.22, 10.96, 13.30 µM), respectively, providing clear proof that the respective position of the groups to influence the activity is necessary to impart activity to the new derivatives.

Shah et al. [97] also studied the anti-proliferative and cytotoxic effects of *J. regia* leaf extracts (methanol and aqueous extracts) at different concentrations on growth inhibitions of cell lines of mice melanoma (B16F10) and human melanoma (A375). The extract concentrations prepared in this experiment included (0.1, 0.15, 0.2, 0.25, 0.3, 0.35, 0.40, 0.45, and 0.5 mg/mL of extract/mL). The normal lymphocyte cell lines were observed to show negligible sensitivity towards the extracts. The cytotoxic activity was screened for 72 h of treatment to the (B16F10) and (A375) cell lines, and it was revealed that methanolic extracts at different concentrations showed potent activity (cell anti-proliferation) against mice melanoma with IC_50_ = 0.234 mg/mL compared to IC_50_ = 0.304 mg/mL on human melanoma cell lines. Likewise, aqueous extracts of the above-mentioned concentrations also showed good activity with (IC_50_ = 0.298 and 0.350 mg/mL) against mice and human cell melanoma, respectively. Even though both methanolic and aqueous extracts had significant anti-proliferative action against mice and human cancer cell lines, significant activity was detected for methanol extracts generated at the same concentrations in this investigation.

### 3.8. Antifungal Activity

The antifungal activity of aqueous and solvent extracts from leaf and bark helps in medicinal use because these extracts showed a wide range of activity against fungus using various methods such as agar dilution, disc diffusion, agar streak dilution, and the Radish method [98]. The antifungal activity was demonstrated by various research, which includes the major breakthrough contributed by D. Wianowska et al. by comparison of the antifungal activity of different extracts of *Juglans regia* cultivars and the major compound juglone. This study compares the antifungal effects of juglone and extracts from walnut green husks of the cultivars Lake, Koszycki, UO1, UO2, and non-grafted against plant pathogenic fungi such as *Alternaria alternata*, *Rhizoctonia solani*, *Botrytis cinerea*, *Fusarium culmorum*, *Phytophthora infestans*, as well as *Ascosphaera apis*. The data obtained demonstrate that the antifungal activities of the extracts can be modulated by their other constituents and are not always dependent on the antifungal activity of juglone. This enables us to draw the conclusion that juglone is not the only element in walnut green husk extracts that inhibits mycelial growth. It was discovered that phenolic compounds were in charge of the extracts’ activity and that they could change juglone’s antifungal properties [99]. Similarly, the activity was demonstrated by Hubert Sytykiewicz et al. [100] by conducting a study to assess the antifungal efficacy of four extract fractions (methanolic, ethyl acetate, alkaloid, and hydrolyzed methanolic) derived from *Juglans regia* (L.) leaves against pathogenic *Candida albicans* strains. One reference strain (*C. albicans* ATCC 900et 29) and 140 isolates from various biological samples, including skin lesions, sputum, urine, and feces, were used to test the yeasts. The highest anticandidal activity was found in the methanolic extract from walnut leaves, followed by the alkaloid fraction, which had a slightly lower antifungal efficacy. Etyl acetate and hydrolyzed methanolic preparate had the lowest levels of growth rate inhibition for the examined fungal pathogens [101].

Similar antifungal activity was assessed using the disc diffusion method with extract concentrations of 100, 200, and 300 g/mL/disc, using the standard ketoconazole (40 g/mL/disc). Selective fungistatic activity was demonstrated by the extracts against some species. Different levels of inhibitory activity were present in all extracts against all types of fungi. The study revealed that acetone and chloroform extracts significantly inhibited the growth of *Alternaria alternata* and *Trichoderma virens*, respectively. Moreover, methanolic extract demonstrated significant activity against *Aspergillus niger* [102]. The other studies which have been carried out by analyzing the different extract of *Juglans regia* and the antifungal activity have demonstrated that, no matter the type of extract evaluated for the antifungal activity, juglone is the primary component of walnut green husk extracts which demonstrated the maximum antifungal activity. However, the activity varied depending on the composition of other vital extract constituents and the kind of fungal infection that was treated [100,103].

### 3.9. Cardiovascular Activity

Walnuts have been found to have high quantities of omega-3 and omega-6 polyunsaturated fatty acids (PUFA). While some studies have connected omega-6 PUFA to an enhanced proinflammatory vascular response, the bulk of investigations have indicated that these components have no deleterious consequences on human cardiovascular health. It has been also discovered that in non-hyperlipidemic persons, eating walnuts regularly (30–100 g/day) lessened the cardiovascular risk factors [104]. Consuming nuts on a regular basis has been linked to a lower risk of both fatal and non-fatal myocardial infarction. According to epidemiological studies, compared to people who never consumed nuts, those who consumed nuts five or more times per week had a 50% lower risk of coronary heart disease [105,106]. Green walnut hull extract suppressed protein secretion and thrombin-induced platelet aggregation by 50% in an in vitro investigation, with no deleterious effects on platelets at a dosage of 50 mg/mL. Walnut green hull extract’s antiplatelet activity is most likely due to its polyphenolic components and antioxidant capabilities. As a result, it can be regarded a thrombotic disease candidate as well [107].

### 3.10. Brain Enhancing Activity

A healthy functioning brain requires sufficient amounts of water, vitamins (such as folate, thiamine, vitamins B6, and B12), lipoic acid, lutein, and n-3 fatty acids for proper and improved functioning. Walnuts are high in n-3-linolenic acid (ALA), as well as a variety of other potentially neuro-regenerative substances such as phenolic acid (ellagic acid), gamma tocopherol (vitamin E), folate, melatonin, flavonoids, and a plant-based omega-3 fatty acid. It is worth noting that walnuts placed in second place among 1113 foods that were tested for antioxidant levels [108]. Memory is the retaining process of a learning experience across time. By delivering the right stimuli, a single memory can be retrieved. Polyphenols have been demonstrated to exhibit properties which influence the key neuronal signaling pathways in memory and learning. Based on obesity, hypercholesterolemia, and oxidative stress, walnut polyphenolic extracts improved learning by 42% and memory in hypercholesterolemic rats [109]. A walnut diet containing 6% walnut oil protected male rats from neurotoxicity caused by the chemotherapeutic drug cisplatin, according to another study. The results demonstrated that walnut administration enhanced cognitive and motor skills, implying that including walnut in one’s diet may be beneficial in combating chemotherapy-induced motor and cognitive dysfunction [108]. It was also revealed that walnuts enhanced learning skills, locomotor activity, memory, anxiety, and motor coordination in the transgenic mice model against Alzheimer’s disease by 6–9 percent [110]. The above studies which were carried in vivo have clearly opened a new path of the research to demonstrate the same effect on humans while making sure the earlier conclusion and the ample number of dosages are delivered hand in hand alongside with the safety of the subject.

## 4. Toxicity Activity on Plants and Animals

After conducting toxicology research on humans and animals, it was discovered that in the acute oral toxicity trial, Wistar female rats were given several doses of methanolic extracts from 10 to 5000 mg/kg of *J. regia* septum for 14 days. In a sub chronic study, the Wistar rats were fed the extract orally at a rate of 1000 mg kg^−1^ for 28 days. No harmful effects or deaths were observed for the extract even at a concentration of 5000 mg kg^−1^. Further in the study, there were no notable morphological or histological alterations in the studied tissues [111]. According to the Yang et al. [112], walnut polyphenols increased immune function by lowering oxidative stress. Tropical walnut use has been also linked to skin irritation and discoloration in many cases reported in the past, clearly indicating the fact that taking an excess diet of walnut fruit is also harmful to human health [113]. This fact was also well supported by developing enormous blisters and skin discoloration in a 65-year-old woman after she consumed 15 kg of walnuts in three days [114]. It was also revealed that walnut aqueous extract decreased the cyclophosphamide toxicity and protected metabolizing and antioxidant enzymes at the time of treatment on the woman. The goal of the current study was to determine whether walnut extract can reduce the toxicity of the anticancer drug cyclophosphamide (CP) while also protecting against the disruption of enzymes that help the body break down drugs and fight free radicals [113]. Human erythrocyte forward scatter was significantly reduced after 24 h of exposure to juglone (5 µM). Juglone (1–5 µM) markedly raised the proportion of annexin V-binding cells. Juglone (5 µM) markedly increased the amount of ceramide at the erythrocyte surface and decreased the amount of erythrocyte adenosine triphosphate (ATP). Juglone stimulates suicidal erythrocyte death or eryptosis, at least in part, by increasing the abundance of ceramides (lipid molecule), depleting energy, and activating protein kinase (PKC) [114]. Walnut’s detrimental effects on animals, notably horses, have also been reported. Moreover, walnut heartwood can induce laminitis, an inflammatory disease in horses. As a result, the black walnut extract model has been created to study the many parameters connected to horse laminitis [115].

To investigate and validate the much promising study in relation to toxicity caused by the walnut excess diet, Iwamoto et al. investigated the effects of walnut consumption on serum lipids and blood pressure in Japanese subjects to determine whether it would also be beneficial as a component of the Japanese diet. In a crossover design, they randomly assigned 20 men and 20 women to one of two mixed natural diets for four weeks each. Both diets adhered to the typical Japanese diet (reference diet) and included the same foods and macronutrients, with the exception that the walnut diet provided 12.5% of its energy from walnuts (43–57 g/d) (offset by lesser amounts of fatty foods, meat, and visible fat). When comparing the walnut diet to the reference diet, total cholesterol levels were 0.21 mmol/L lower for women (*p* < 0.01) and 0.16 mmol/L lower for men (*p* = 0.05). When they followed the walnut diet, the LDL cholesterol levels were 0.18 mmol/L lower for the men (*p* = 0.13) and 0.22 mmol/L lower for the women (*p* < 0.01). The walnut diet also decreased the concentration of apolipoprotein B and the ratio of LDL to HDL cholesterol (*p* < 0.05) [116].

## 5. Conclusions

The current review addresses the most prominent published research on *J. regia* L. in medical sciences, the plant’s traditional and modern scientific applications, as well as scientific validation of the stated biological activity in vivo and in vitro. Synthetic triazole derivatives suggested in the manuscript can be modified to create new potent molecules that can be tested for a variety of biological activities such as anticancer, antidiabetic, antibacterial, and many others with promising results that will stand in the research gap. Furthermore, various extracts (aqueous or methanolic) from walnut leaves, flowers, or fruits might be utilized in concentration-dependent ways in a new study to make improvements in the relevant field. To the best of our knowledge, we have not reported any deceptive activity after working on our own green fruit and leaf extract of the plant, as well as manipulation of other work in the same field. However, more human trials are needed to determine all *J. regia* Linn extracts’ much anticipated and promising capabilities and activities.

## Figures and Tables

**Table 1:** List of compounds in *Juglans regia* and their pharmacological or biological activities.

Compound	Class	Structure	Biological Activity	References
Gallic Acid	Phenolic	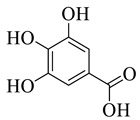	Anti-inflammatory	[5,6]
Protocatechuic acid	Phenolic	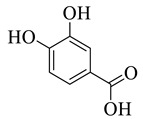	Anti-inflammatory and antiapoptotic activities.	[7]
Ferulic acid	Phenolic	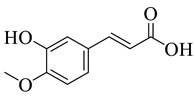	Anticancerous	[8]
Sinapate	Phenolic	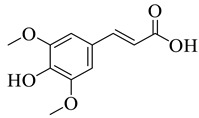	Antioxidant, Antimicrobial	[9]
Protocatechuic acid derivative	Phenolic	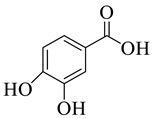	Anti-inflammatory	[10]
Ellagic acid	Phenolic	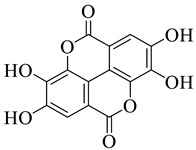	Cytotoxic, Anti-proliferative	[11]
p-hydroxybenzoic acid	Phenolic	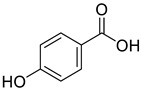	Antimicrobial	[12]
p-coumaric acid	Phenolic	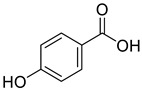	Antioxidant	[13]
Quercetin 3-galactosid	Phenolic	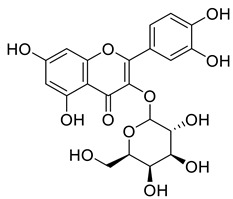	Antimicrobial, Antioxidant	[14]
Galactose	Phenolic	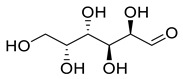	Anticancer, Antidiabetic	[15]
Pentose	Phenolic	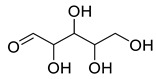	Amino acid synthesis	[16,17]
Arabinose	Phenolic	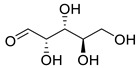	Antimicrobial	[18]
Xylose	Phenolic	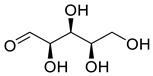	Antimicrobial	[19,20]
Rhamnose	Phenolic	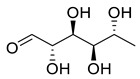	Antioxidant, Antimicrobial	[21]
Juglone	Phenolic	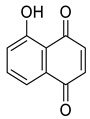	Antibacterial, Anticancer, Antioxidant	[22]
Caffeic acid	Phenolic	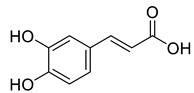	Antioxidant, Anti-inflammatory and Anticarcinogenic activity	[23]
Vannilic acid	Phenolic	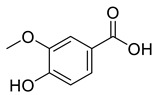	Antibacterial, Antioxidant	[24]
Quercetin	Flavonoid	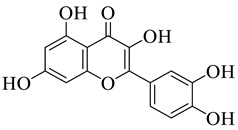	Analgesic, Antibacterial, Antiviral	[25]
Myricetin	Flavonoid	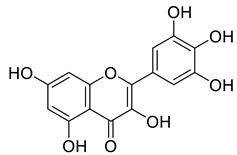	Anticancer, Antidiabetic, Antibacterial, Antiviral	[26,27]
Kaempferol	Flavonoid	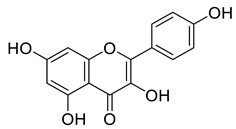	Acute and chronic inflammation	[28]
Apigenin	Flavone	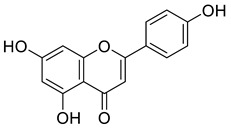	Anti-inflammatory, Antioxidant, Neuroprotective	[29]
Luteolin	Flavone	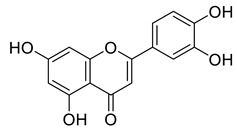	Anticancer, Anti-inflammatory	[30]
Daidzein	Isoflavonoid	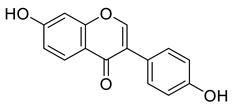	Neurobiological activities	[31]
Naringenin	Flavanone	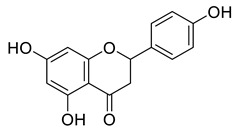	Antiviral, Antibacterial, Antioxidant	[32]
Hesperetin	Flavonone	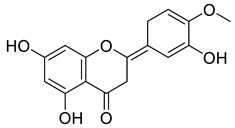	Scavenging activity and Cardioprotective activity	[33,34]
Gallocatechin	Flavanol	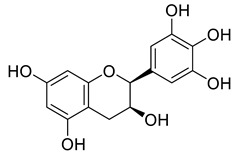	Antioxidant, Antitumor	[35]
Sitosterol	Steroid	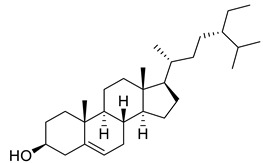	Anticancer, Antidiabetic, Antimicrobial	[36]
Stigmast-5-en-3β,7α- diol	Steroid	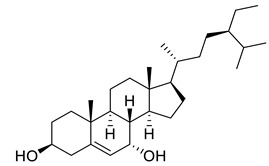	Antimicrobial	[37]
Campesterol	Steroid	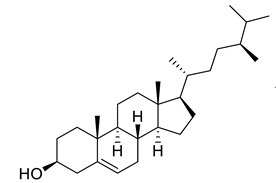	Anti-inflammatory, Anticancer, Antidiabetic	[38]
Stigmasterol	Steroid	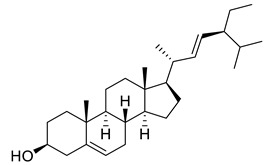	Anticancerous Dyslipidemia, diabetes and metabolic syndrome	[39]
Oleanic acid	Tereponoid	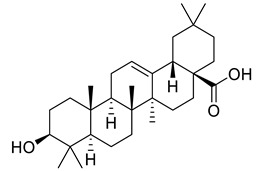	Dyslipidemia, diabetes, and metabolic syndrome	[40]
3-alpha-Corosolic acid	Tereponoid	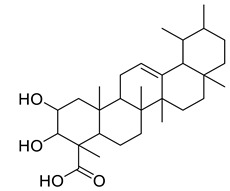	Antidiabetic, Anti-obesity, Anti-inflammatory	[41]
Urosolic acid	Tereponoid	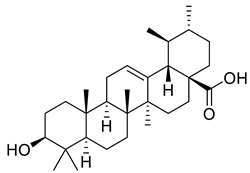	Anti-inflammatory, Anticancer, Antidiabetic, Antioxidant	[42]
3-Epikatonic acid	Tereponoid	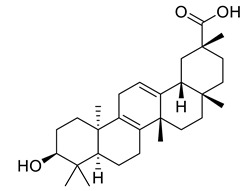	Cytotoxic	[43]

**Table 2:** Phytochemical analysis of different extracts, their effect, dosage, and model organism or experimental reference screened.

Pharmacological Effect	Extract	Model/Experimental-Reference	Dosage-Range	References
Antibacterial	Hull extract of ethanol, ethyl-acetate and water	*E. coli, B. subtilis, K. aerogenes, S. Aureus*	5 mg/mL	[52,53]
Antibacterial	Aqueous leaf extract	Gram-positive and Gram-negative bacteria	0.1 mg/mL (MIC)	[54,55]
Antibacterial	Essential oil and components	Gram-positive and Gram-negative bacteria	Wide range	[56]
Antioxidant	Ethyl acetate, butanol, ether, and aq. Extracts of (kernels, husk, and leaves)	NR	125 µL	[57]
Antioxidant	Leaf extract (ethanol and water)	NR	34.5 and 56.4 µg/mL	[58,59]
Analgesic and anti-inflammatory	Aqueous and ethanolic extract of leaf	Wistar rats, human arthritis	250, 500, 1000, 1500 mg/kg	[60]
Antiviral	Methanolic extract	HSC, polio virus and SINV	2 mg/mL	[61]
Antidiabetic	Leaf extract	Human type-ii diabetic patients	Wide range	[62]
Anticancer	Juglone	Intestinal carcinogenesis	Wide range	[63]
Anticancer	Methanol and aqueous extract of leaf	Mice melanoma (B16F10) and human melanoma (A375) cell lines	Wide range	[64]
Anticancer	Juglone and modified juglone	(NCl-H322 and A549) Lung cancers	Wide range	[65]
Anticancer	Chloroform leaf extract	Human oral and breast cancer cell lines	Wide range	[66]

## Data Availability

Not applicable.
